# Assessing the Efficacy of Various Pain Management Methods in Orthodontic Debonding: A Randomized Clinical Trial

**DOI:** 10.7759/cureus.88483

**Published:** 2025-07-22

**Authors:** Shaik Farhatulla, Inuganti Ranganayakulu, Anisha Valli Anumallasetty, Neelapala Rohini, Nachu Manojna, RSVM Raghu Ram

**Affiliations:** 1 Orthodontics and Dentofacial Orthopaedics, GSL Dental College and Hospital, Rajahmundry, IND

**Keywords:** debonding, elastomeric wafer, finger pressure, stress relief, vibration anaesthesia, visual analog scale

## Abstract

Introduction: Orthodontic treatment improves smiles and confidence, but pain during procedures often leads to high dropout rates, making effective pain management essential. Debonding, which marks the completion of treatment, can be particularly painful. Although advanced pain relief methods such as analgesics, local anesthesia, ultrasound techniques, transcutaneous electrical nerve stimulation, and nitrous oxide sedation are available, they are often costly, time-consuming, and may affect patient compliance. In contrast, simple and cost-effective techniques like finger pressure, elastomeric wafers, stress relief, and vibration anesthesia offer practical alternatives to reduce pain and enhance patient satisfaction. However, there is limited literature comparing the effectiveness of these methods.

Objectives: To evaluate the pain experienced during orthodontic debonding using finger pressure, elastomeric wafers, stress relief, and vibration anesthesia, and to assess the effectiveness of these pain control methods with respect to age and gender variations.

Methodology: The descriptive cross-sectional study adhered to Strengthening the Reporting of Observational studies in Epidemiology (STROBE) guidelines, involving 140 (61 males and 79 females) orthodontic patients aged 18-30 years, who had undergone fixed orthodontic treatment with 0.022” x 0.028” slot McLaughlin, Bennett, and Trevisi (MBT) prescription metal brackets were randomly divided into four groups. A single debonding plier is used, employing elastomeric wafer, finger pressure, stress relief, and vibration anesthesia methods. Visual Analog Scale (VAS) scores were recorded after debonding. Statistical data was analysed using IBM SPSS Statistics for Windows, Version 20 (Released 2012; IBM Corp., Armonk, New York, United States).

Results: Upper jaw pain scores differed significantly among groups, favouring the elastomeric wafer group (p<0.001) with a low score. Vibration anesthesia group resulted in higher mean pain scores, particularly in the upper regions (p<0.001). In the lower jaw, the elastomeric wafer group had lower mean pain scores, with no significant difference from finger pressure group (p=0.514). Overall, the elastomeric wafer group demonstrated significantly lower mean pain scores, with no gender-based differences observed.

Conclusions: Elastomeric wafer and finger pressure methods were effective in orthodontic pain management during debonding, along with a stress relief method to a certain level. Vibration anesthesia method is comparatively less effective for pain reliving method while debonding, particularly in anterior regions. The study found no gender differences in the pain scores across all groups.

## Introduction

Orthodontic treatment is widely recognized for its ability to enhance a patient's smile, boost self-esteem, and improve overall quality of life [1]. However, pain and discomfort during treatment are common, leading to significant patient dropout rates. Studies reveal that 80-95% of patients abandon treatment due to pain intolerance [2]. Therefore, effective pain management during orthodontic procedures is crucial for improving patient experience and ensuring treatment success.

Pain is defined by the International Association for the Study of Pain as “an unpleasant sensory and emotional experience associated with actual or potential tissue damage” [3]. Pain during orthodontic treatment is influenced by physical, psychological, genetic, hormonal, and social factors [4]. Tooth morphology also matters, with anterior teeth being more sensitive due to thinner cortical boundaries and greater force [5].

In orthodontics, pain measuring scales such as Visual Analog Scale (VAS) and Pain Catastrophizing Scale (PCS) are common, but PCS has limitations such as subjectivity, cultural bias, and negativity. However, the VAS is frequently favoured due to its simplicity, versatility, and effectiveness [6,7]. Several orthodontic procedures, such as separator placement, arch wire placement, and debonding, are associated with pain. Debonding is crucial as it marks treatment completion, and minimizing discomfort enhances patient experience. Orthodontic discomfort can be alleviated using methods like anesthetics, cyclooxygenase (COX)-2 inhibitors, lasers, vibratory stimulation, ultrasound, and cryotherapy, though these are often costly and time-consuming [8]. Simpler, cost-effective techniques such as finger pressure, elastomeric wafers, stress relief, and vibration anesthesia are commonly used to reduce pain and improve patient comfort. Thus, this study aims to assess the effectiveness of various pain management approaches, including finger pressure, stress relief, elastomeric wafers, and vibration anesthetic device, during orthodontic debonding, thereby enhancing patient comfort and overall satisfaction with orthodontic treatment.

## Materials and methods

This descriptive cross-sectional study was conducted in accordance with the Strengthening the Reporting of Observational studies in Epidemiology (STROBE) guidelines for reporting observational studies and adhered to the Declaration of Helsinki for research involving human participants. The study protocol received approval from the Clinical Trial Registry of India (CTRI/2025/06/089211).

All patients whose records were used were given complete information about the study and provided written informed consent. The study included patients who visited the Department of Orthodontics, GSL Dental College and Hospital for treatment and met the inclusion criteria. Participants were assigned to one of four groups using simple randomization through a computer-generated random number table. Allocation concealment was maintained using sequentially numbered, opaque, sealed envelopes (SNOSE). Due to the nature of the interventions, blinding of participants was not possible. However, outcome assessment was single-blinded - the person recording pain scores was not aware of the group assignments.

Patients aged 18 to 30 years who were able to understand, provided they had no history of taking medication (specifically painkillers, corticosteroids, or antiviral drugs) within the last 24 hours. Participants were required to be undergoing fixed orthodontic treatment, specifically using McLaughlin, Bennett, and Trevisi (MBT) prescription 0.022-inch metal brackets with a miniature single-mesh base (3M Unitek Gemini Metal Twin Brackets, 3M India Limited, Bangalore, Karnataka), along with 0.019 × 0.025-inch finishing arch wires, for a minimum of two months.

Individuals with a history of surgical interventions or craniofacial abnormalities, such as cleft lip and palate, were excluded from the study. Additionally, those with missing, heavily restored, or root canal-treated teeth were not eligible. Patients experiencing ongoing periodontal issues, mainly greater than grade I mobility, were also excluded. Lastly, individuals undergoing treatment with mini screws or those with brackets found to be debonded at the time of debonding were excluded from the study.

A total of 140 patients were randomly allocated into four groups: elastomeric wafer, finger pressure, stress relief, and vibration anesthesia, with 35 participants in each group. The study sample consisted of 79 female and 61 male participants. The sample size was determined using G*Power version 3.1.9.2 (Heinrich-Heine-Universität Düsseldorf, Germany). The calculation was based on a Cohen’s effect size of 0.8 (considered a large effect), a type I error probability (α) of 5%, and a power of 90%, with an allocation ratio of 1:1 between groups. These parameters were chosen based on findings from a preliminary pilot study, which indicated a substantial difference in pain perception across the different debonding methods. This sample size was considered adequate to detect clinically meaningful differences between the interventions with high statistical power, minimizing the risk of type II error.

Patients who were approaching the final stage of appliance withdrawal after completing fixed appliance treatment were selected for the debonding procedure. To reduce the potential influence of anxiety on pain reporting, all participants were provided with standardized reassurance and procedural explanation before the debonding procedure. Additionally, the use of a single operator and consistent clinical environment across all groups helped minimize variability related to anxiety or operator-related bias.

To maintain procedural consistency, the debonding procedure was performed by a single experienced operator using the same instrument and standardized technique across all groups. The Waldent Orthodontic Bracket Remover Plier 10/124 (Waldent Innovations Pvt. Ltd., New Delhi, India) was used uniformly for all patients. The plier was carefully positioned in an occlusogingival direction between the bracket base and the tooth surface, and brackets were removed by applying controlled, gentle pressure [9]. The use of a single trained operator, uniform instrumentation, and a standardized method helped minimize operator-related variability and procedural errors, thereby enhancing the reliability of the study outcomes.

Debonding methods

Various debonding methods were utilized to manage pain are as follows:

(i) Elastomeric Wafer Group

**Figure 1:**
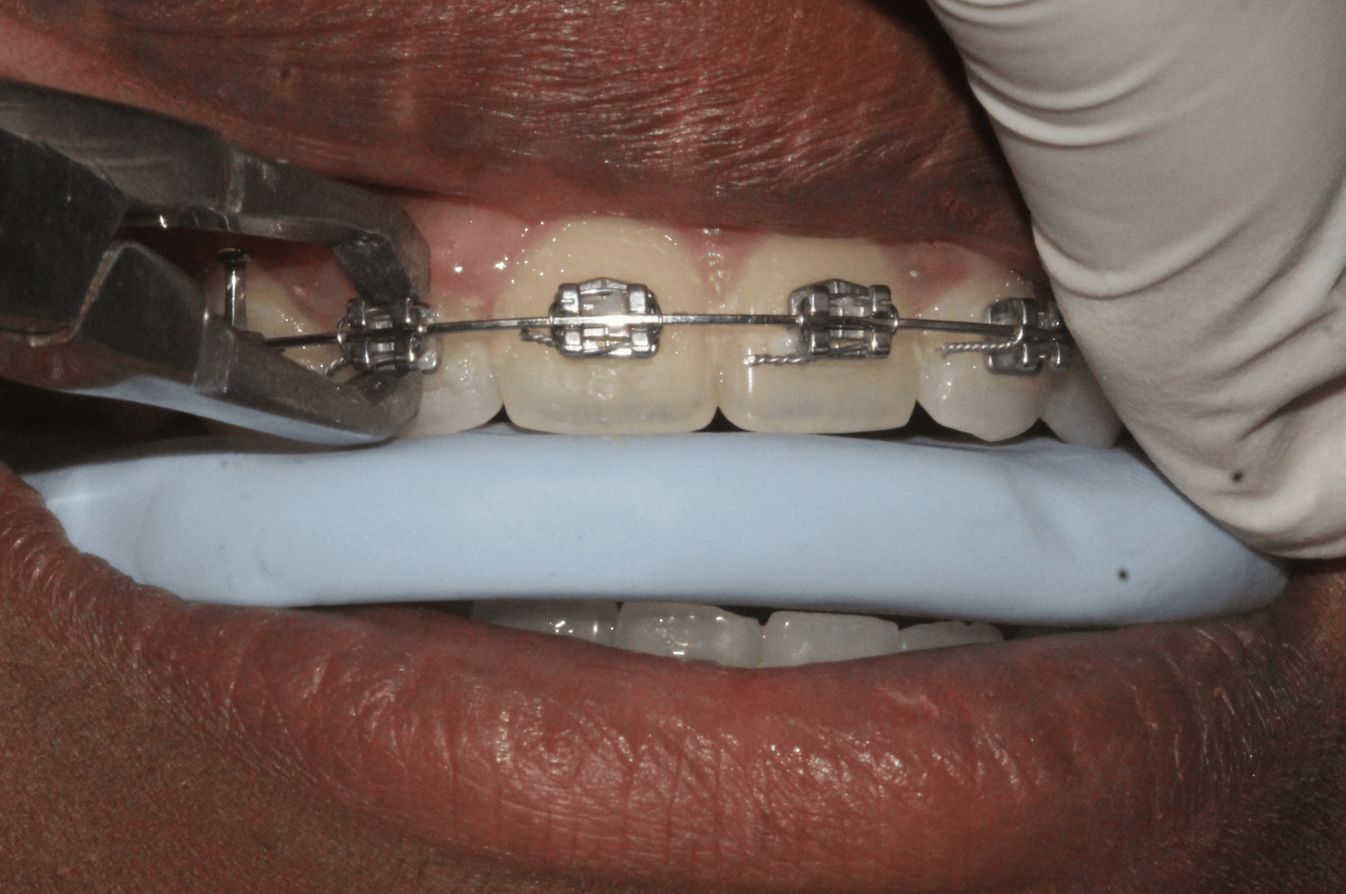
Elastomeric wafer method

A heavy-body silicone printing impression material, Dentsply Aquasil Soft Putty Regular Set (Dentsply India Private Limited, Karnataka, India), was used to create an arch-shaped bite raiser known as a wafer bite, approximately 5-6 mm thick. Arch forms were fabricated based on patient casts using the symmetric measurement grid for reference. The elastomeric wafer was sterilized by immersion in a 2% active glutaraldehyde solution for 10 minutes prior to use. It was then placed between the dental arches, and patients were instructed to bite down firmly during bracket debonding (Figure 1).

(ii) Finger Pressure Group

**Figure 2:**
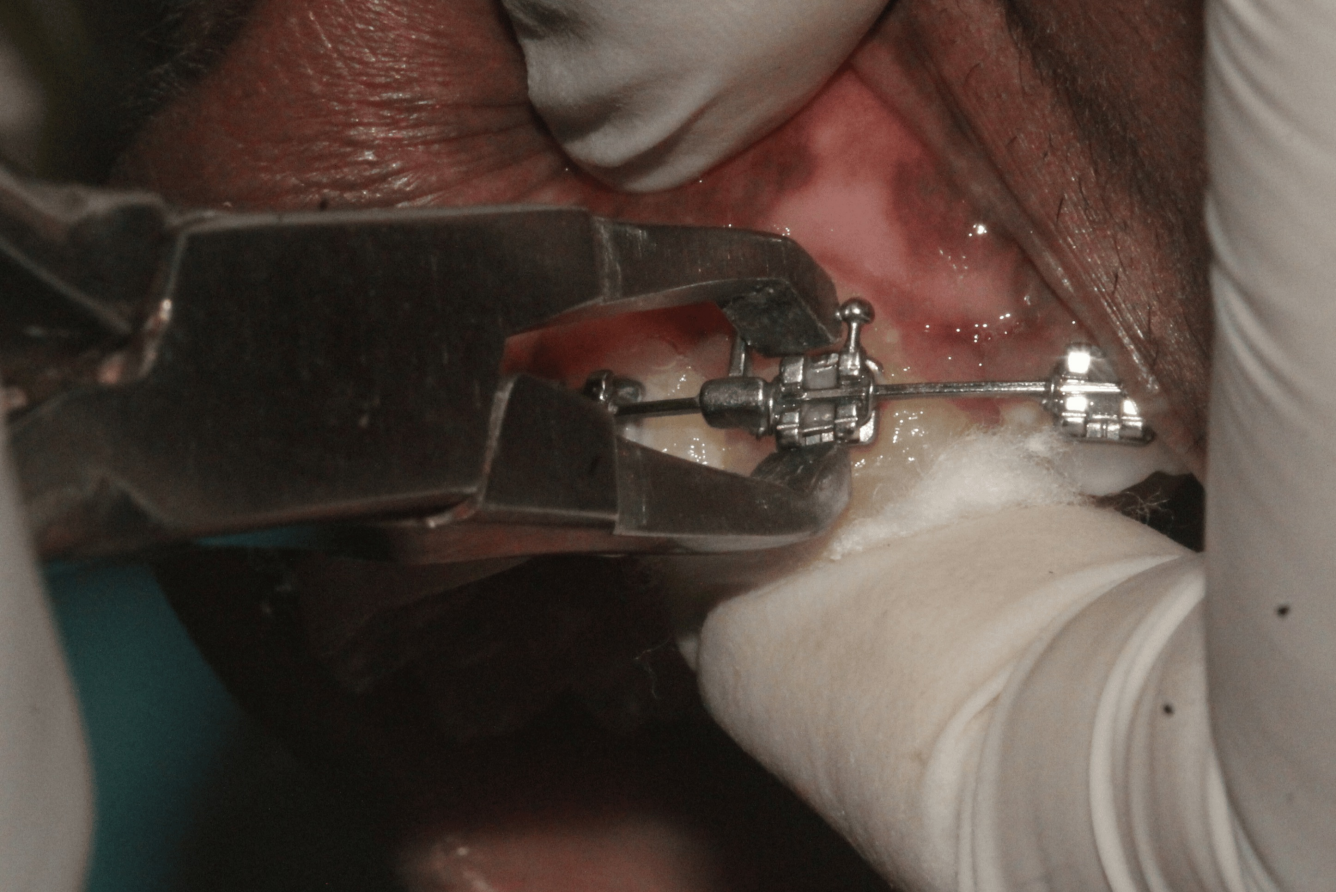
Finger pressure method

During the debonding of each bracket, the operator applied finger pressure from the incisal or occlusal surface of the tooth in a gingival direction using their thumb. To minimize the effects of occlusal morphological variations, a cotton roll was placed under the thumb (Figure 2).

(iii) Stress Relief Group

**Figure 3:**
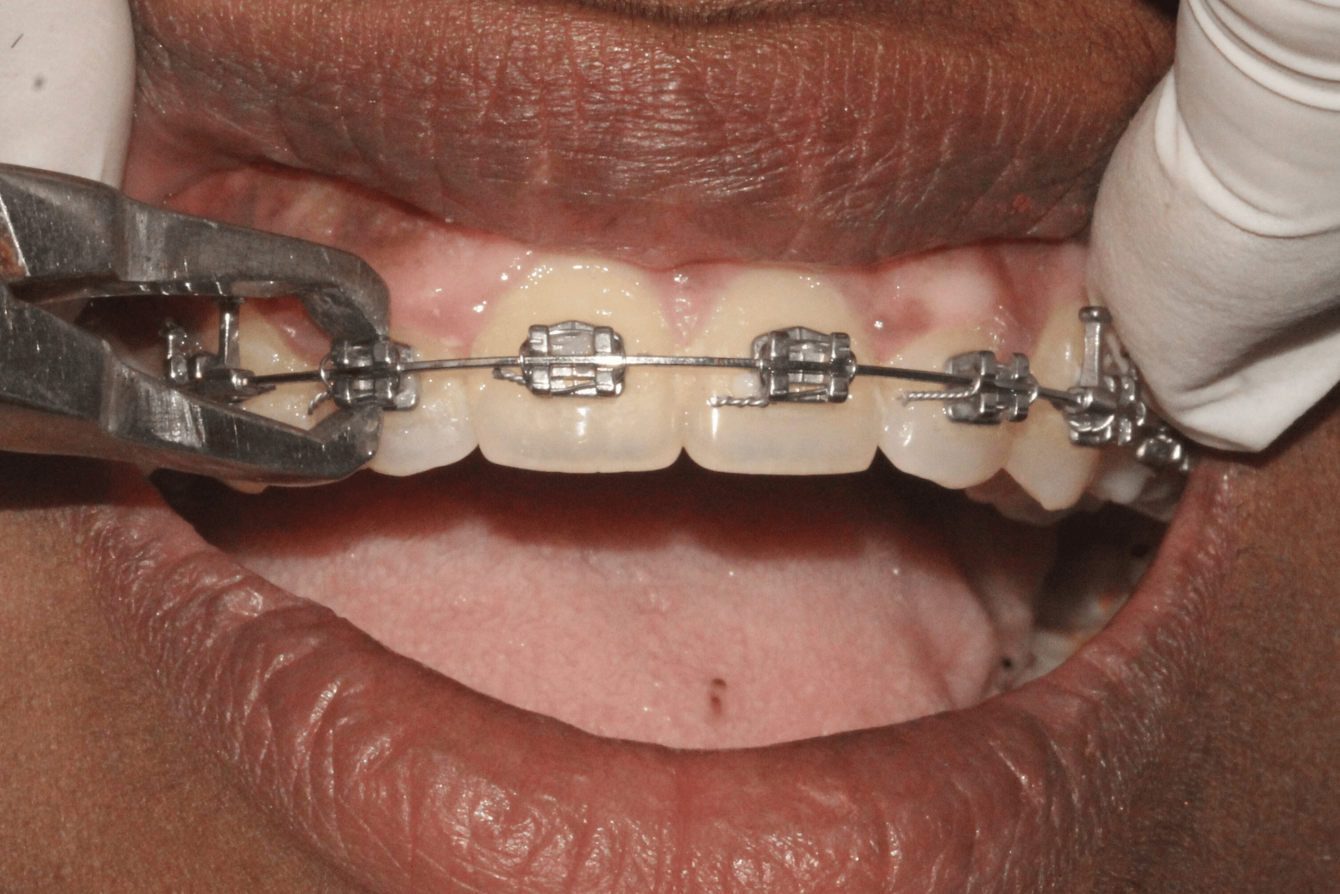
Stress relief method

This method supplemented regular debonding practices. Patients were advised not to occlude their teeth during the process. To reduce anxiety, reassurance was provided, explaining that debonding would not lead to significant discomfort or pain (Figure 3).

(iv) Vibration Anesthetic Device Group

**Figure 4:**
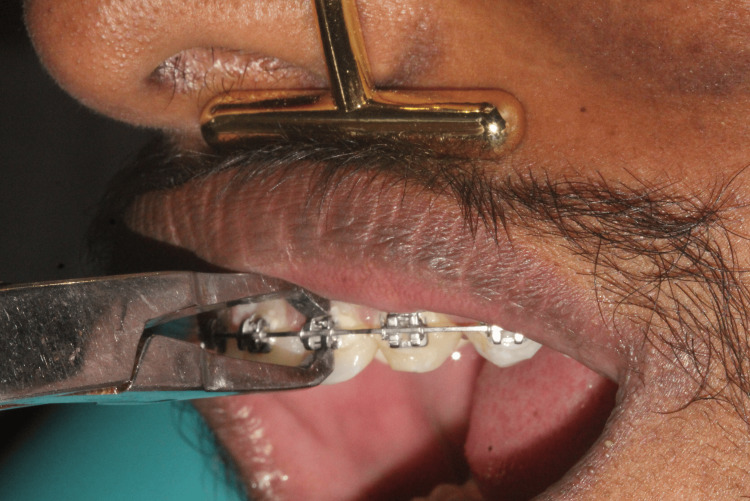
Vibration anesthetic device method

A vibration device (24K Gold Energy Beauty Bar Electric Vibration Facial Massage Roller, Kaya International, New Delhi, India), which produces 6000 vibrations per minute at 100Hz, was applied extra-orally at the sulcus line, 5 mm above the root apex of the teeth designated for debonding. The device was activated and positioned five seconds prior to debonding and continued to operate throughout the procedure. It was removed from the site five seconds after debonding (Figure 4).

The archwire remained in place while the brackets were debonded with a torquing movement, one at a time, from upper right to upper left and from lower right to lower left by a single operator with the same debonding pliers and the same operator. Immediately after debonding, the operator asked patients to report their pain using the VAS method.

A 100-mm VAS was prepared for each tooth, where a score of 0 indicated “no pain,” with increasing scores up to 100 representing greater pain. Patients were instructed to record their pain scores on this scale following debonding. Before the debonding procedure, participants were informed about the study’s objectives and were instructed that they would need to assess pain intensity using a VAS for each sextant, including upper right (UR), upper front (UF), upper left (UL), lower right (LR), lower front (LF), and lower left (LL).

## Results

Table 1 compares the mean ages of the study groups, showing a mean age of 22.09 ± 4.08 years in the vibration anesthetic device group, while the stress relief, finger pressure, and elastomeric wafer groups had mean ages of 21.89 ± 4.23, 23.17 ± 5.97, and 23.17 ± 5.97, respectively. The differences in mean age among the groups were not statistically significant.

**Table 1:** Comparison of mean age between the study groups One-way analysis of variance (ANOVA); p ≤ 0.05 considered statistically significant.

Group	N	Mean	Standard deviation	Standard error	95% confidence interval for mean		F-value	P-value
					Lower bound	Upper bound		
Vibration anesthetic device	35	22.09	4.083	0.690	20.68	23.49	0.627	0.599
Stress relief	35	21.89	4.234	0.716	20.43	23.34		
Finger pressure	35	23.17	5.978	1.010	21.12	25.22		
Elastomeric wafer	35	23.17	5.978	1.010	21.12	25.22		

Table 2 presents the gender distribution across the groups, with no significant differences observed.

**Table 2:** Comparison of gender distribution between the study groups Chi-square test; p ≤ 0.05 considered statistically significant.

Group	Sex		Total	Chi-square value	P-value
	Male	Female			
Vibration anesthetic device	15	20	35	0.3191	0.956
Stress relief	14	21	35		
Finger pressure	16	19	35		
Elastomeric wafer	16	19	35		

Table 3 summarizes inter-group comparison of pain scores across all groups and regions, showing the vibration anesthetic device with the highest mean scores and elastomeric wafers with the lowest.

**Table 3:** Inter-group comparison of pain scores One-way analysis of variance; * p ≤ 0.05 considered statistically significant.

Group	Mean	Standard deviation	F-value				P-value	Mean	Standard deviation	F-value				P-value	
	Upper right							Lower right							
Vibration anesthetic device	32.57	15.967	9.93				<0.001*	26.00	10.627	11.72					<0.001*
Stress relief	25.14	12.919						17.14	13.410						
Finger pressure	20.86	15.024						12.86	11.523						
Elastomeric wafer	15.43	9.500						10.86	10.675						
	Upper front							Lower front							
Vibration anesthetic device	32.29	16.104	11.25			<0.001*		43.14	15.102	17.17			<0.001*		
Stress relief	28.00	15.301						33.14	9.933						
Finger pressure	19.14	11.472						24.86	12.919						
Elastomeric wafer	16.57	7.253						24.00	12.414						
	Upper left							Lower left							
Vibration anesthetic device	29.14	17.719	6.74		<0.001*			28.86	14.506	12.72		<0.001*			
Stress relief	18.00	12.788						20.86	15.024						
Finger pressure	18.00	12.078						12.00	10.792						
Elastomeric wafer	16.00	10.901						12.86	11.523						
	Upper total							Lower total							
Vibration anesthetic device	94.00	26.701	32.05	<0.001*				96.29	27.449	33.27	<0.001*				
Stress relief	71.43	21.164						71.14	21.112						
Finger pressure	58.00	19.373						49.71	19.018						
Elastomeric wafer	48.00	13.890						49.14	23.056						

Table 4 presents multiple pairwise comparisons for pain scores across various methods and sextants. The vibration anesthetic device consistently recorded the highest pain scores, while elastomeric wafers reported the lowest. Pairwise comparisons revealed significant differences between groups, especially between the vibration anesthetic device and other techniques. Stress relief and finger pressure showed moderate scores, with elastomeric wafers being the most effective in reducing pain. Statistically significant differences were noted in most comparisons.

**Table 4:** Multiple pairwise comparisons for pain scores Tukey’s post hoc tests; * denotes significance; p ≤ 0.05 considered statistically significant.

Reference group	Comparison group	Mean difference	P-value	Mean difference	P-value
		Upper right		Lower right	
1	2	7.429	0.106	8.857	0.009*
	3	11.714	0.002*	13.143	<0.001*
	4	17.143	<0.001*	15.143	<0.001*
2	3	4.286	0.552	4.286	0.414
	4	9.714	0.017*	6.286	0.112
3	4	5.429	0.342	2.000	0.889
		Upper front		Lower front	
1	2	4.286	0.516	10.000	0.007*
	3	13.143	<0.001*	18.286	<0.001*
	4	15.714	<0.001*	19.143	<0.001*
2	3	8.857	0.026*	8.286	0.036*
	4	11.429	0.002*	9.143	0.016*
3	4	2.571	0.842	0.857	0.992
		Upper left		Lower left	
1	2	11.143	0.005*	8.000	0.056
	3	11.143	0.005*	16.857	<0.001*
	4	13.143	0.001*	16.000	<0.001*
2	3	0.000	1.000	8.857	0.027*
	4	2.000	0.927	8.000	0.056
3	4	2.000	0.927	-0.857	0.993
		Upper total		Lower total	
1	2	22.571	<0.001*	25.143	<0.001*
	3	36.000	<0.001*	46.571	<0.001*
	4	46.000	<0.001*	47.143	<0.001*
2	3	13.429	0.038*	21.429	0.001*
	4	23.429	<0.001*	22.000	0.001*
3	4	10.000	0.189	0.571	1.000
		Overall score		-	
1	2	48.000	<0.001*		
	3	82.571	<0.001*		
	4	93.143	<0.001*		
2	3	34.571	<0.001*		
	4	45.143	<0.001*		
3	4	10.571	0.514		

Table 5 compares pain scores based on sex across different regions, showing no statistically significant differences (p > 0.05) between males and females in any region, total scores, or overall scores.

**Table 5:** Comparison of the pain scores based on sex Independent sample t-test (two-tailed); p ≤ 0.05 considered statistically significant.

Region	Sex	Sample	Mean	Standard deviation	t-value	P-value
Upper right	Male	61	22.95	14.983	-0.382	0.702
	Female	79	23.92	14.799		
Upper front	Male	61	26.39	14.723	1.730	0.084
	Female	79	22.15	13.929		
Upper left	Male	61	19.67	13.780	-0.446	0.66
	Female	79	20.76	15.002		
Upper total	Male	61	69.18	29.286	0.499	0.61
	Female	79	66.84	24.989		
Lower right	Male	61	16.89	12.321	0.142	0.891
	Female	79	16.58	13.386		
Lower front	Male	61	30.82	14.640	-0.329	0.744
	Female	79	31.65	14.973		
Lower left	Male	61	17.21	15.070	-1.010	0.312
	Female	79	19.75	14.320		
Lower total	Male	61	64.59	28.318	-0.698	0.491
	Female	79	68.10	30.970		
Overall score	Male	61	133.77	48.172	-0.126	0.90
	Female	79	134.81	48.750		

## Discussion

The current clinical trial aimed to evaluate the effectiveness of various pain management methods during orthodontic debonding. To mitigate the effects, the study focused on a specific age range of 18 to 30 years, similar to Gupta et al. [10]. Bavbek et al. noted that females and those with pain catastrophizing tendencies typically report higher pain levels, prompting an examination of gender distribution in the study groups, which revealed no significant differences between male and female participants. To control bias, a single debonding plier was used, and all procedures were done by one operator following Bavbek et al. [11]. The present study investigates new strategies to enhance patient comfort during debonding. Elastomeric wafers were chosen for their ease of use and potential for chair-side fabrication [12]. The finger pressure technique was highlighted for its simplicity and effectiveness in pain control, offering advantages like cost-effectiveness and minimal technique sensitivity. Vibration anesthesia was noted for its superior pain control, particularly in the scalp (plasma rich protein) procedures and local anesthesia applications [13,14]. Acknowledgment of the role of cognitive behaviour in stress relief reinforces the selection of these methods.

In the present study, the mean VAS scores reported by the elastomeric wafer group closely align with Karobari et al. findings but differ in the lower anterior region [6]. However, Mangnall et al. documented higher scores potentially due to age differences among participants [15]. The finger pressure and stress relief groups reported higher mean VAS scores compared to the findings of Karobari et al. [6]. This significant pain variation is attributed to the operator's pressure during debonding and is influenced by patient anxiety and the operator's counseling approach, respectively. In the upper jaw, vibration anesthesia provided the least pain relief, while stress relief was more effective anteriorly. Finger pressure and elastomeric wafers showed no significant differences. In the lower jaw, vibration anesthesia was more effective except in the anterior, where stress relief varied, and finger pressure matched elastomeric wafers.

Overall, the vibration group exhibited significantly higher pain scores, especially in the anterior region, aligning with Mangnall et al. findings [15]. Stress relief had higher scores than finger pressure and elastomeric wafers, but lower than vibration. Koyama et al. highlighted that patient trust and positive expectations can reduce pain [16]. The comparison between the finger pressure group and the elastomeric wafer group showed no significant differences in pain scores. In this study, VAS scores were highest in the anterior teeth, which are more sensitive due to smaller surface area and lower sensory threshold (1 g) compared to posterior teeth (5-10 g), as noted by Karobari et al. [6]. Similar findings have been reported in previous studies, further supporting the role of anatomic factors in pain perception [15,17]. Intrusive force reduces pain by easing stress on the periodontal ligament [18]. Finger pressure is a cost-effective, time-efficient method for pain control, especially in the lower arch [11].

The study faced methodological issues, including not accounting for diurnal variation in post-debonding pain and lacking standardized force application during debonding across groups. Additionally, the mixed sample of extracted and non-extracted cases added variability to the population. Future research should examine diurnal variation in post-debonding pain and implement standardized force application techniques during debonding. Additionally, studies should consider restricting case selection to either extracted or non-extracted premolars to reduce confounding factors and enhance focus.

## Conclusions

Elastomeric wafer and finger pressure were effective in orthodontic pain management during debonding, along with stress relief to a certain level. Vibration anesthesia was comparatively less effective as a pain-relieving method during debonding, particularly in the anterior region. The study found no gender differences in the pain scores across elastomeric wafer, finger pressure, stress relief, and vibration anesthesia groups.
